# Microenvironmental stimuli for proliferation of functional islet β-cells

**DOI:** 10.1186/2045-3701-4-12

**Published:** 2014-03-04

**Authors:** Hanan Alismail, Sha Jin

**Affiliations:** 1 Department of Bioengineering, College of Engineering, University of Arkansas, Fayetteville, AR 72701, USA; 2 Department of Bioengineering, Thomas J. Watson School of Engineering and Applied Sciences, State University of New York in Binghamton, Binghamton, NY 13902, USA; 3 College of Applied Medical Sciences, King Saud bin Abdulaziz University for Health Sciences, National Guard - Health Affairs, P.O. Box 2490, Riyadh 11426, Kingdom of Saudi Arabia

**Keywords:** Islets of Langerhans, β-cell proliferation, Diabetes, Insulin, Mitogen

## Abstract

Diabetes is characterized by high blood glucose level due to either autoimmune destruction of islet β-cells or insufficient insulin secretion or glucose non-responsive production of insulin by β-cells. It is highly desired to replace biological functional β-cells for the treatment of diabetes. Unfortunately, β-cells proliferate with an extremely low rate. This cellular property hinders cell-based therapy for clinical application. Many attempts have been made to develop techniques that allow production of large quantities of clinically relevant islet β-cells *in vitro*. A line of studies evidently demonstrate that β-cells can proliferate under certain circumstances, giving the hopes for generating and expanding these cells *in vitro* and transplanting them to the recipient. In this review, we discuss the requirements of microenvironmental stimuli that stimulate β-cell proliferation in cell cultures. We highlight advanced approaches for augmentation of β-cell expansion that have recently emerged in this field. Furthermore, knowing the signaling pathways and molecular mechanisms would enable manipulating cell proliferation and optimizing its insulin secretory function. Thus, signaling pathways involved in the enhancement of cell proliferation are discussed as well.

## Introduction

Pancreatic β-cell is considered as one of the most important cell types in the islet of Langerhans in pancreas. It is responsible to insulin production in response to blood glucose level. The result of either insulin deficiency or sugar level irregulated insulin secretion is diabetes mellitus [1]. Type I and II diabetes are two common types of this disease. Type I diabetes (T1D) results from the autoimmune destruction of β-cells, making the body incapable of maintaining normoglycemia in these patients. In type II diabetes (T2D), either the body does not produce enough insulin due to a decrease in functional β-cell mass, or the insulin secretion does not correlate to glucose levels in the blood. Diabetes induces other diseases, including heart disease and stroke, high blood pressure, kidney disease, and blindness. The Centers for Disease Control and Prevention estimated that 25.8 million people of the U.S. population are having this disease in 2011 [2]. World Health Organization suggested that the global number of diabetic patients would reach 300 million and being the 7^th^ leading cause of death by the year 2030 [3]. Current treatment for T1D includes insulin supplement by either tablet, injection, or organ transplantation [4]. However, organ transplantation causes complications regardless the scarcity of donors and histocompatibility matching issues [5]. On the other hand, medical treatment for T2D is fairly limited. Driven by the urgent need to treat patients suffering from diabetes, intensive research efforts have been made to create biologically functional islet tissues that can be used to replace diseased islets and to regenerate a healthy tissue for the realization of cell-based therapy.

One of the main challenges remains in adult pancreatic β-cell therapy is the extremely low proliferation rate which is approximately 0.1 ~ 0.3% a day in aged adult animal and minimum replication capacity in adult human [6]. Fortunately, β-cell mass expansion can occur in early postnatal life, pregnancy, and animal models which were genetically modulated [7-9]. Thus, it is important to fully understand the molecular mechanisms that allow enhancing the pancreatic β-cell proliferation, so that cells can be cultured in an environment suitable for *in vitro* production. Inducers of β-cell proliferation can be classified to extrinsic and intrinsic path. Extrinsic mitogens include: glucose, amino acids, insulin like growth factors, prolactin (PRL), placental lactogen (PL), glucagon-like peptide-1 (GLP-1), growth hormone, hepatocyte growth factor (HGF), epidermal growth factors, transforming growth factor (TGF), and extracellular matrix (ECM) [10-12]. The intrinsic factors include cyclins, cyclin dependent kinases, and cyclin dependent kinas inhibitors [13]. This review focuses on the most important extrinsic mitogens and signaling pathways that are involved in the process of β-cell proliferation. The review also overviews advanced approaches and applications in the field of islet β-cell expansion and biological functionalization.

## Native β-cells and their surroundings

**Figure 1:**
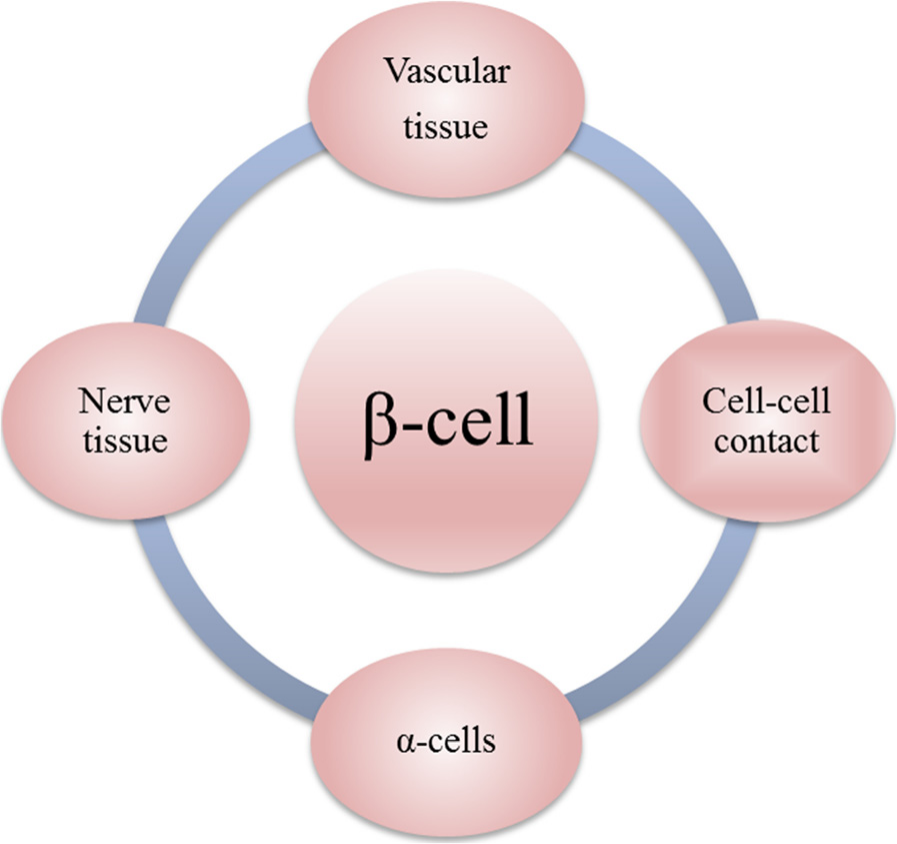
Summary of β-cell interaction with pancreatic environment.

Islets of Langerhans are comprised of five types of cells: α, β, δ, ε, and PP-cells. These cells work as a micro organ to maintain glucose homeostasis. β-cell is the most abundant and important cell in islets, which senses the circulating glucose level in the blood and responses glucose level by secreting insulin accordingly [14]. β-cell receives regulation signals from a pancreatic and non-pancreatic environment that promote its function and proliferation [14]. As diagramed in Figure 1, first of all, a dense vascular network exists within the islets facilitates efficient oxygen and insulin secretion. β-cells cross interact with the endothelial cells of the capillary network through the vascular basement membrane. β-cells secret vascular endothelial growth factor to promote the vascular development, whereas the endothelial cells produce a basement membrane rich with laminin to support the insulin gene expression and secretion from β-cells and further β-cells proliferation [15]. Second, cell-cell contacts between β-cells, through several transmembrane receptors, have a great impact on insulin gene expression and glucose stimulated insulin secretion (GSIS) [16]. Third, β-cells interact with α-cells in reciprocal secretion to maintain glucose homeostasis [17]. Fourth, islets are rich with neurons from sympathetic and parasympathetic nervous system. Interaction between β-cells and parasympathetic neurons activates specific receptors to induce GSIS, whereas sympathetic neurons inhibit insulin secretion as a part of the physiological glucose homeostasis [18] (Figure 1). Moreover, β-cells receive signals from non-pancreatic tissues such as: liver, bone, fat, and gut, endocrine cells of the intestine [14]. These cells secrete integrins which bind to a G-coupled receptor on the β-cell surface to stimulate the insulin secretion and β-cell proliferation [19]. In the process of islet isolation all of these vascular and nerve connections are destroyed by enzymatic digestion of the pancreas and islet purification through a density centrifugation, which could be the major cause of malfunction of β-cell and low survival after isolation procedures [20,21]. Motivated by the need of creating an optimal niche for β-cell expansion, biologically functional materials and signaling molecules for creating a niche that can support cell expansion both *in vitro* and after transplantation have been explored. The details are discussed as follows.

## Extrinsic mitogens

### Glucose

Glucose is one of the important regulators in β-cell proliferation, since the primary function of β-cell is to lower blood glucose level by insulin secretion. Evidence indicating the role of glucose in the β-cell proliferation has been reported in several studies both in *in vitro* and *in vivo*. For example, glucose is a key nutrient for cell growth in fetal and neonatal β-cell [22], insulinoma cell lines [23], primary islets [24], and human β-cells [25]. *In vivo* glucose infusion subjected to diabetic mice and rats result in increase in β-cell mass ultimately [26-28].

**Figure 2:**
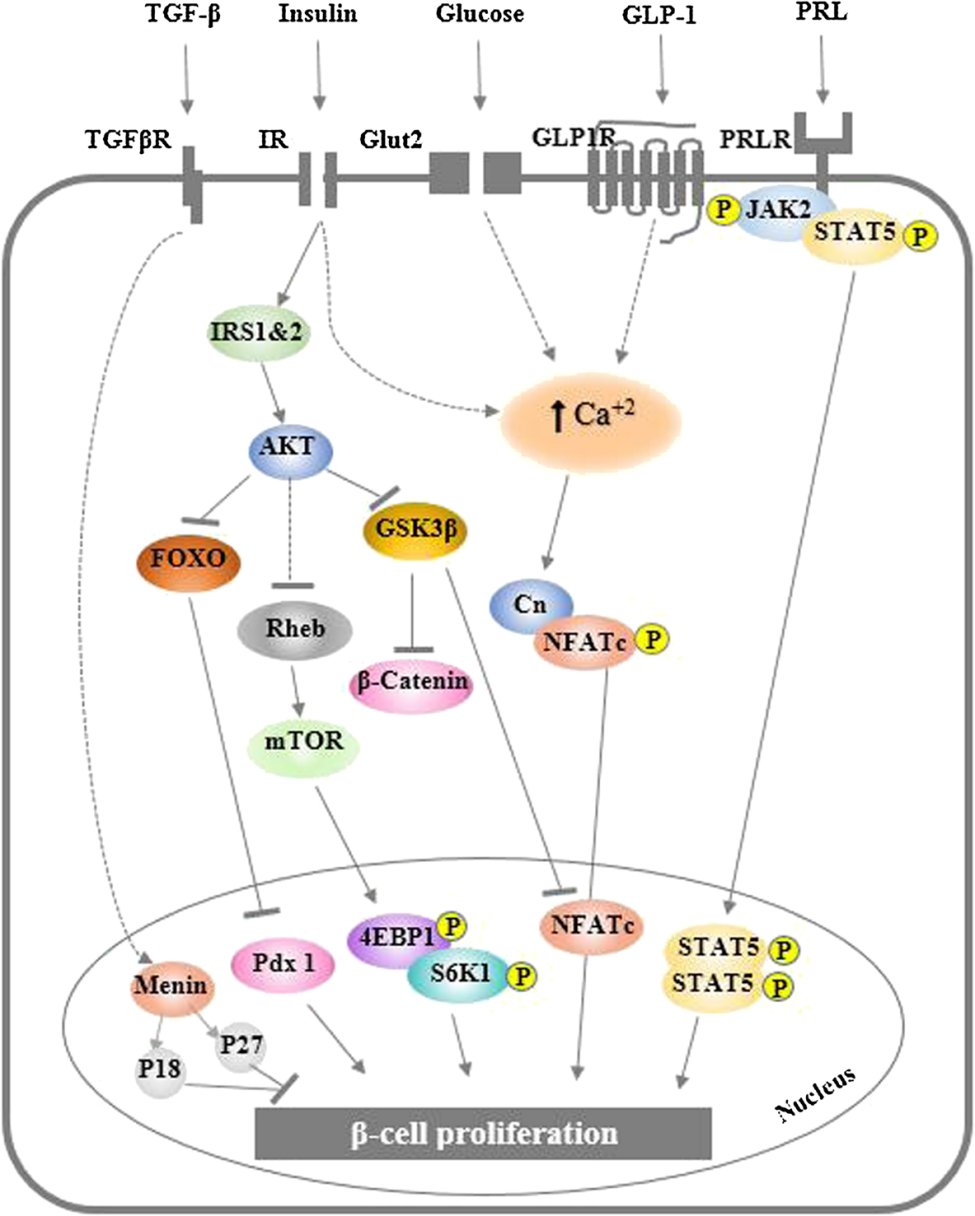
**Schematic of β-cell extrinsic mitogens and pathways regulating β-cell proliferation.** Abbreviations: Cn, Calcineurin; GLP1R, GLP-1 receptor; IR, insulin receptor; PRL, prolactin; PRLR, prolactin receptor; TGFβR, transforming growth factor-β receptor.

The signaling pathways which are correlated glucose with β-cell quantity, proliferation, and apoptosis have been extensively investigated. Several pathways revealed to be involved are: (1) insulin autocrine effect, (2) calcium signaling, and (3) TSC2/mTOR inhibitory signaling pathway [29] (Figure 2). *In vitro* studies demonstrated that glucose induces intracellular signaling molecules such as phosphatidylinositol 3-kinase (PI3K), protein kinase B (PKB), glycogen synthase kinase-3 (GSK-3), extracellular signal-regulated kinase (ERK)1/2, and mammalian target of rapamycin (mTOR), as well as insulin receptor substrate 2 (IRS2) [30,31]. Activation of insulin receptor leads to the activation of the Akt signaling pathway which is considered to be one of the main pathways of β-cell proliferation [32,33]. Moreover, this activation is down regulated by mTOR signaling triggered by an increase in ATP production and leads to the subsequent inactivation of AMP kinase (AMPK) [15]. Finally, the calcium signaling pathway has also a significant remark through cornering, the only calcium regulated phosphates. Study on an investigation of the importance of the pathway has been conducted by deleting the calcineurin regulatory subunit, calcineurin b1 (Cnb1). This study results in reduced β-cell proliferation and developing age related diabetes. The correction for this defect was made by expressing the active nuclear factor of activated T cell cytoplasmic 1 (NFATc1), which is a downstream transcription factor in this pathway [34]. Moreover, activation of transcription factors cAMP response element-binding protein (CREB) and serum response factor (SRF) can improve β-cell growth rate through glucose/calcium pathway [35,36].

Although the moderate glucose elevation causes an increase in β-cells growth and survival, prolonged exposure to high concentration of glucose is the main cause of β-cell deterioration and apoptosis. This condition is called glucotoxicity, which is caused by several of mechanisms that are not fully understood [37,38]. A line of studies found a number of transcriptional regulators that are sensitive to the level and duration of glucose and insulin gene expression such as MafA, NFAT [39,40], somatostatin transcription factor-1 (STF-1), pancreatic and duodenal homeobox 1 (Pdx-1), and the insulin control element (ICE) [41]. It was shown that these proteins are expressed in the presence of 0.8 mM glucose and in a prolonged exposure to a high level of 11.1 mM glucose [37]. In addition, MafA protein level is able to restore insulin expression in β-cell lines along with the present of prolonged high glucose level [42]. High glucose concentrations have also shown to inactivate AMP protein kinase (AMPK) in the β-cell and result in impaired GSIS due to lipid accumulation which leads to the deterioration of β-cell function [43-45].

Another set of studies revealed that glucose toxicity may lead to oxidative stress in organelles such as in the endoplasmic reticulum and mitochondria [46,47]. Increase in oxidative stress causes a reduction binding of Pdx1 and MafA to insulin gene in pancreas, which in turn results in defective insulin gene expression and hormone secretion [42,48]. It is clear that the reactive oxygen species (ROS) activate stress-induced pathways, including nuclear factor kB (NF-κB), and c-Jun N terminal kinase (JNK) pathways [49,50]. The subsequent JNK signaling event leads to the inactivation of IRS-1 by its phosphorylation on Ser307 [51]. A recent study suggested that hypoxia is another reason causing malfunction of β-cells. Expression of transcription factor hypoxia-inducible factor (HIF) plays an adverse role in β-cell function [52]. Under the exposure to a high glucose level, an alteration in the profile of β-cell gene expression occurs, including a switch from aerobic to anaerobic glycolysis that leads to impaired GSIS and glucose intolerance. This was investigated through the deletion of the regulatory protein von Hippel-Lindau (VHL) protein for controlling the degradation of HIF which reduces the cellular oxygenation level and causes hypoxia. VHL/HIF oxygen-sensing mechanisms play a critical role in glucose homeostasis through decreasing islet oxygenation level and negatively impact β-cell function [53,54].

Furthermore, chronic hyperglycemia leads to excessive accumulation of Ca^2+^ in the cytosol which is a proapoptotic signal that induces β-cell dysfunction and destruction [55]. Other potent apoptosis pathways were shown to be related to interleukin-1 β which inhibits β-cell function and promotes Fas-triggered apoptosis in part by activating the transcription factor NF-κB during the autoimmune process of T1D pathogenesis [56]. However, knowledge of the signaling pathway involved in chronic hyperglycemia remains elusive. In particularly, the targets and the downstream effects of glucotoxicity have not been completely elucidated.

### Growth factors and signaling pathways

#### Insulin growth factor (IGF)

Expressions of insulin growth factor I (IGF-I) and II (IGF-II) and their receptor (IGFR) were found in different stages of pancreatic development. In fact, their expressions act as signal for stimulating β-cell proliferation [57]. IGF-I and IGF-II are able to enhance β-cell proliferation in rat islets and insulinoma cell lines *in vitro*[58]. Interestingly, the over-expression of IGF-I results in enhanced proliferation of β-cells in transgenic mice but not the size of islets, while the over-expression of IGF-II leads to abnormal islet morphology with enlarged irregular shape [59,60].

Studies also demonstrated that insulin receptor (IR) is a stimulator for β-cell proliferation [61], where the Akt and MAPK signaling pathways are involved [62]. Reduction in the IR by up to 80% in mouse β-cells MIN6 leaded to reduction of growth rate, suggesting that insulin plays a crucial role as a growth factor for this insulinoma cell line [63]. In addition, the effect of IR substrate 1 and 2 (IRS1 & 2) has also been explored as they are modulators in the insulin/IGF signaling cascade as indicated in Figure 2. Heterozygous mutation in IRS1/IR in mice showed approximately 400-fold increase in circulating insulin in parallel to severe insulin resistance with striking hyperplasia in the β-cell mass by 40-50-fold [64]. In contrast, animal models deficient in IRS-2 showed abnormalities such as low proliferation, low Pdx-1 expression, small islet size, and increase in apoptosis [65]. Moreover, IRS-2 over-expression is associated with glucose stimulation in insulinoma cells. It exhibits a synergistic increment in β-cell proliferation in glucose/IGF-1 induced manner *in vitro*[66]. Another study reported that IRS-2 controls other growth promoting mutagens such as exendin-4, which protects β-cells from human islet amyloid polypeptide-induced cell damage [67,68]. All of these studies suggested that the downstream signaling of insulin and IGF receptors are essential for maintaining β-cell mass and proliferation.

#### Akt/PKB signaling pathway

Akt is also known as protein kinase B (PKB), is proposed to be a crucial modulator of IRS-2-mediated signal in β-cells (Figure 2). Deficiency in AKT2 has shown a negative impact on β-cell proliferation, which provides an evidence of its importance in the signaling cascade [12]. On the contrary, AKT2 over expression induced β-cell proliferation via enhancing resistance to apoptosis and improving insulin secretion [32,33]. Some downstream targets such as forkhead transcription factor 1 (FoxO1), glycogen synthase kinase 3 beta (GSK3β), and the mammalian target of rapamycin (mTOR) are down-regulated by the Akt signaling pathway and Akt functions have been described elsewhere [69-71] (Figure 2).

Pdx-1, also known as insulin promoter factor 1 (IPF1), has been investigated as an ultimate transcription factor of Akt signaling, result of its phosphorylation and nuclear inclusion plays an important role in β-cells proliferation and function [72]. However, it is still unknown how Pdx-1 regulates β-cell proliferation. In addition, Pdx-1 can restore β-cells function in IRS2 knocked out mice, suggesting that the dysregulation of Pdx-1 by IRS2 is directly related to the development of T2D [73].

There are multiple evidences for the inversely correlation between the forkhead transcription factor FoxO1 and the Pdx-1 expression. A group of studies showed several mechanisms in which FoxO1 can antagonize Pdx-1. FoxO1 suppression can be done through the competition binding of forkhead box protein A2 (FoxA2) protein to Pdx-1 promoter region and restoring Pdx-1 expression level [74]. This was further affirmed by the expression of Pdx-1 in β-cells which contain cytoplasmic FoxO1 but not nuclear FoxO1 [74]. A recent study also confirmed that FoxO1 inhibits β-cell neogenesis but it is required for the maintenance of insulin secretion under metabolic stress [75]. In addition, FoxO1 can lead to a nuclear exclusion of Pdx-1 in oxidative stressed β-cell as a suppression mechanism [76]. However, it is unclear the type of molecules that are stimulated by Pdx-1 and the mechanism of these molecules correlating with Pdx-1 to induce β-cell proliferation.

#### Prolactin, placental lactogen, and HGF

Several other growth factors, such as PRL and PL, can also enhance β-cell proliferation rate both *in vitro* and *in vivo*. Prolactin as well as PL are highly expressed during pregnancy and showed to be involved in increasing β-cells mass [77]. Injection of PRL or PL leads to higher β-cell growth in experimental rodent [78]. Moreover, transgenic mice over-expressing PL demonstrated a higher β-cell growth rate associated with hyperinsulinemia [78]. In contrast, mice lacking PRL receptor reduce β-cell mass [79]. The PRL receptor belongs to the cytokine receptor family that is involved in the JAK/STAT signaling pathway [80]. Thus, binding of PL and PRL molecules to PRL receptor triggers β-cells proliferation through signaling pathway JAK2/STAT5 (Table 1).

**Table 1:** Summary of extrinsic factors and their downstream signaling pathways involved in β-cell proliferation

**Extrinsic factors**	**MAPK**	**Akt**	**Ca**^**+2 **^**signaling**	**Jak/Stat**	**Smad**	**β-catenin**
Glucose	√		√			
Insulin/IGF-1		√	√			
PRL/PL				√		
HGF	√	√	√			
TGF					√	
GLP-1/GIP	√	√	√			√

HGF is a mesenchyme derived growth factor involved in proliferation, migration, and differentiation of several types of tissues [81]. Deletion of HGF demonstrated glucose intolerance and impaired GSIS. A protective effect against apoptosis and increase of proliferation was exhibited in induced dysfunction mice injected with exogenous HGF gene [82]. HGF binds to c-Met receptor and activates MAPK and PI3K/Akt pathways which are responsible for β-cell proliferation [83]. Hence, HGF seems to be an attractive potential target for therapy.

#### Transforming growth factor-beta (TGF-β)

Transforming growth factor (TGF) family is an important regulator in the pancreas development and function. It has two classes of polypeptide growth factors, TGF-α and TGF-β. They have been implicated in the pathogenesis of cancer, autoimmune disease, and diabetes. Several studies have reported that the impaired TGF level can lead to the onset of both T1D and T2D [84]. Impaired TGF-β is related to the progression of T1D in non-obese diabetic (NOD) mice. The cause of diabetes in these mice was autoimmune destruction of islets resulting insulin deficiency [85]. An adenoviral expression vector encoding TGF was transected to the NOD mouse islet cells. The experimental result suggested a protection to the NOD mouse islet cells from apoptosis and immune distraction and delay in diabetes occurrence for 22 days compared to the only 7 days vector transfected control [86]. Microarray on isolated intact human islets incubated in low and/or high glucose revealed a highly regulated TGF signaling in the human islets. This suggests that TGF is involved in the glucose metabolism and β-cell function as well [87,88].

### Incretins

Incretins are a group of hormones that lead to an increase in the amount of insulin released from the β-cells. GLP-1 and glucose-dependent insulinotropic polypeptide (GIP) are the two types of incretin hormones that have been well investigated. They are secreted from the intestine upon glucose ingestion to stimulate insulin secretion. These two hormones have shown to be involved in increasing β-cell proliferation and decreasing cell apoptosis [89]. GLP-1 infusion into glucose-intolerance rats caused an increase in the β-cell mass. Likewise, an increase in the β-cell size and neogenesis was observed in mice treated with GLP-1. Moreover, GLP-1 has an anti-apoptotic effect in freshly isolated human islets [90]. Buteau and his co-workers found that GLP-1 enhances the binding of NF-κB transcription factor to two anti-apoptotic genes: inhibitor of apoptosis protein-2 and Bcl-2, resulting in augmentation of the expression of the anti-apoptotic proteins [7]. Therefore, GLP-1 has been approved by the FDA for being used for T2D treatment [91]. GIP has also shown to be a synergistic mitogen inducer with glucose and a pleiotropic growth factor for insulin-producing on INS-1 β-cells [92]. Indeed, GIP is strictly glucose dependent and it does not show any effect during a low blood glucose level. Thus, GIP seems to act as a blood glucose stabilizer with inverse glucose-dependent effect on pancreatic insulin [93]. In addition, the binding of GIP-1 to its G-protein couple receptor (GLP1R) activates downstream targets including cAMP and PKA, intracellular calcium, and Pdx-1, leading to Pdx-1 expression [94-96] (Figure 2). For instance, mouse islet β-cell treated with GLP1R can activate PI3K/Akt signal pathway and trigger a significant increase in IRS2 [97]. On the contrary, inhibition of both c-SRC and EGFR suppresses GLP1R-mediated PI3K pathway in INS-1 cells [98]. Recently, GLP-1 was found to be able to induce β-cell proliferation by increasing the β-catenin nuclear content and increasing cyclic D1 expression [99]. Table 1 summarizes the extrinsic factors and the downstream signaling pathways involved in β-cell proliferation.

### Extracellular matrix

Adult human islets are surrounded by an incomplete capsule constituted from a single layer of fibroblasts and collagen fibers. Additional matrix protein is attached to this capsule and known as peri-insular basement membrane. Mechanical and chemical signaling interactions between cells and ECM are known to regulate several philological aspects including: survival [100], proliferation [101], and insulin secretion in islets [102]. In living tissue, cells synthesize ECM components and deposit them to form a niche. A niche not only affects the tissue composition and mechanical properties, but also determines cellular fate. As aforementioned that islet β-cell can rarely proliferate *in vitro*. Thus, many attempts have been made to find out niches required for β-cell expansion. In a recently study, human islet cells were cultured in two ECM environments: rat ECM (804G) and bovine corneal endothelial ECM (BCEC) in the presence of GLP-1 analogue, liraglutide. It was observed that there is approximately 0.082 ± 0.034% proliferation of islet β-cells in a liraglutide treated/BCEC culture condition. The result indicates that adult human β-cell proliferation can occur *in vitro* but remains an extremely rare event within an environment of certain ECM and signaling molecule [103]. In another study, fully differentiated human adult insulin-producing β-cell was unable to proliferate *in vitro* regardless of whether or not the presence of human growth hormone (hGH) and the GLP-1 analogue liraglutide. However, hGH and GLP-1 enhanced rat β-cell proliferation [104].

Since an interaction of ECM with integrins triggers an intracellular signaling cascade and modulates the level of gene expressions that control cell behavior [105], effect of integrin on β-cell proliferation has also been explored. Studies suggested that adult human islets are expressing special types of integrins including α3, α5, αv, α6, β1, β3 and β5 [96]. Laminin-5 interacting with α6β1 integrin allows rat β-cell proliferation [106]. Among ligands for the α3β1 integrin, including fibronectin, laminin, collagen I, and collagen IV, only collagen I and IV promote rat INS-1 cell viability and proliferation [107]. Collagen type I, IV, and laminin can increase survival rate of islets after isolation procedures [108]. Nikolova and his co-workers identified laminins as endothelial signals for promoting insulin secretion of β-cells, and this augmentation relies on the interaction between β1 integrin and the laminins [109].

## Tissue engineering approaches

**Figure 3:**
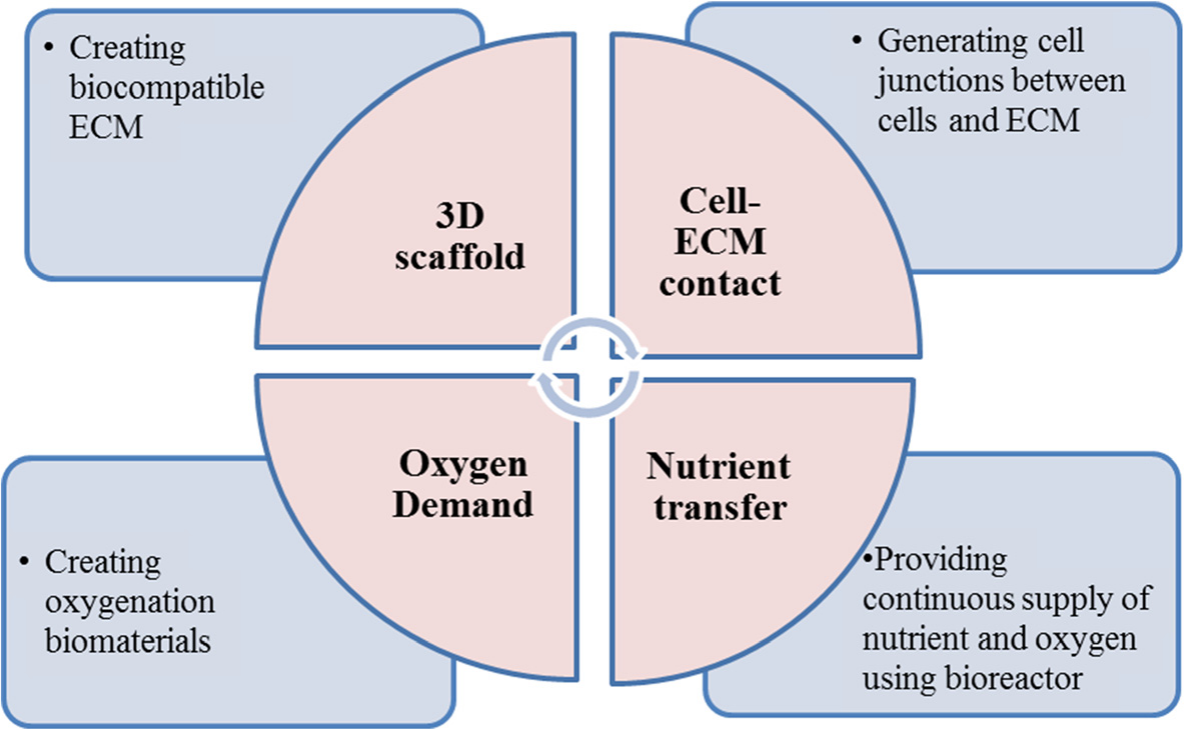
**Tissue engineering approaches for enhancing pancreatic islet growth *in vitro*.**

As mentioned previously, there is a considerable interest to understand the most important regulators and the mechanism that can stimulate the pancreatic islet growth *in vitro*. Tissue engineering approaches have been explored by culturing cells with highly porous scaffold biomaterials to generate a three dimensional (3D) environment for improving the islet growth and survival, as well as normal insulin secretion (Figure 3). A line of studies demonstrated that poly(ethylene glycol) (PEG) hydrogel scaffold can mimic cell-cell communication microenvironment required for insulin-secreting β-cells [110-112]. Bernard and his co-workers developed a PEG hydrogel-based microwell cell culture system using photolithography technique [113]. Mouse β-cells formed aggregates in PEG hydrogels and demonstrated more than 90% cellular viability in a week long culture. Furthermore, aggregated cells showed considerable increase in insulin secretion compared with single cell culture condition [113]. This study indicates that cell-cell adherent junction is one of the paramount factors required for the function of insulin-secreting β-cell. The importance of cell-cell adherent junction for β-cell survival and function is also evidently verified by Kelly group [114]. PEG hydrogel was fabricated to contain collagen type I, collagen type IV, fibrinogen, fibronectin, laminin, and vitronectin, and then used to encapsulate β-cells. β-cell survival was significantly improved in ECM-containing PEG hydrogels compared with in gels without ECM over ten days. Insulin secretion was also enhanced in cells cultured in ECM-containing hydrogels [115]. The PEG/ECM-based scaffolds indeed contribute to the re-establishment of the islets-ECM interaction. Hiscox group developed a device that allows islets to be cultured in between two layers of prevascularized collagen gels. The islets exhibited a higher level of viability and functionality compared to the free islets control [116].

In another engineering approach, encapsulation of islet cells in a 3D scaffold provides protection against the immune cells and its antibodies. However, the diffusion of low molecular weight cytokines through the hydrogels remains to be challenge. To overcome this issue, scaffold surface can be fabricated by coating of PEG-hydrogel with diffusible pro-inflammatory cytokines interleukin (IL)-1β receptor. This modification enabled maintenance of the viability of encapsulated islet cells and function as a glucose-stimulated insulin secretion after the exposure of different cytokines [117]. Another strategy to make better 3D scaffold is to inhibit TNF receptor 1 by scaffolding PEG-diacrylate hydrogels coated with TNF receptor 1. As a result, this modified hydrogel not only preserved islet insulin content, but also reduced mRNA of inducible nitric oxide synthase and IL-6 in pancreases in experimental animals [118]. Nevertheless, impaired oxygen diffusion within a 3D scaffold hinders the wide use of scaffold for islet cell expansion. In particular, normoxia or higher oxygen tension promotes islet β-cell development from progenitor cells and increases β-cell viability [119-121], as β-cells consume large amounts of oxygen during insulin secretion [122]. Studies have shown that islet-like cell aggregates may suffer from hypoxia proportion to the radial distance inward leading to the cell necrosis and apoptosis as well as activation of the anaerobic metabolism [123]. This issue may be overcome by culturing the cell aggregates in an oxygenated system [124]. Recently, an oxygenator made from polydimethylsiloxane (PDMS)/calcium peroxide enhanced the mouse β-cell proliferation and insulin secretion for three weeks under hypoxic culture conditions [120]. The oxygenating strategy is practically promising because islet cells are usually sensitive to chemical compounds such as catalyst or hydrogen peroxide. Alternatively, mouse β-cells were cultured in suspension in a stirred spinner flask to overcome the limitation of nutrient transport in conventional cell culture dish. This bioreactor culture facilitates cell proliferation and enlarges sizes of β-cell aggregates with enhanced responsibility to glucose level and incretin level [125] (Figure 3).

## Concluding remark

Diabetes mellitus become global epidemic diseases in recent years. Especially T2D affects 5.9% of the world’s adult population with limited medication and treatment. This necessitates seeking of novel treatments to control the increase rate of the diseases. Studies have revealed that multiple mitogens or cell-ECM or cell-cell communications can induce biologically functional β-cell proliferation through multitude nutrient and growth factors. Furthermore, understanding β-cell dysfunction and failure mechanism in the development of onset of diabetes is crucial to optimize the treatment options. Due to the extremely low proliferation capability and survival rate of the islet β-cells after isolation procedures, finding out new cell sources for production of clinically relevant β-cells is one of the topics in the field of tissue engineering and regenerative medicine for diabetic treatment. Currently, there are three types of cell sources in the field of regenerative medicine to produce β-cells. They are stem cells, endocrine progenitors, and other mature cells in the pancreas and β-cell itself [126]. Significant progresses have been achieved for each of these strategies. For example, at first glance, human embryonic stem cell (hESC) and its counterpart named induced pluripotent stem cell (iPSC) are considered to be promising sources for β-cell generation *in vitro*. Nevertheless, the differentiation procedure is still under development and investigation. Besides, the risk of teratoma formation *in vitro* remains a major concern if therapeutic β-cells are produced from hESCs or iPSCs. On the other hand, it is possible to produce β-cells from duct-lining, acinar cells [127], or hepatocytes [128], even though it is still under a controversial discussion [129]. It remains unclear which approach will prove ultimately to be successful in clinical applications. To date it remains to be a significant challenge to generate sufficient biologically functional β-cells to replace damaged or malfunctional β-cells. Most likely, the future of diabetes therapies rely on the combination of fabrication of novel constructor with integration of cell, signal molecule, and biomaterial that mimics microenvironment that is suitable for islet β-cell development in the body.

## Competing interest

The authors declare that they have no competing interests.

## Authors’ contributions

HA drafted the manuscript. SJ revised and approved the manuscript. Both authors read and approved the final manuscript.
